# Biodegradable PLA/PBSA Multinanolayer Nanocomposites: Effect of Nanoclays Incorporation in Multinanolayered Structure on Mechanical and Water Barrier Properties

**DOI:** 10.3390/nano10122561

**Published:** 2020-12-20

**Authors:** Tiphaine Messin, Nadège Follain, Quentin Lozay, Alain Guinault, Nicolas Delpouve, Jérémie Soulestin, Cyrille Sollogoub, Stéphane Marais

**Affiliations:** 1 Normandie Univ, UNIROUEN, INSA Rouen, CNRS, PBS, 76000 Rouen, France; tiphaine.messin@gmail.com (T.M.); Lozay.quentin@yahoo.fr (Q.L.); 2 Laboratoire PIMM, Arts et Métiers Institute of Technology, CNRS, Cnam, Hesam Université, 151, Bd de l’Hôpital, 75013 Paris, France; alain.guinault@lecnam.net (A.G.); cyrille.sollogoub@cnam.fr (C.S.); 3 Normandie Univ, UNIROUEN Normandie, INSA Rouen, CNRS, Groupe de Physique des Matériaux, 76000 Rouen, France; nicolas.delpouve1@univ-rouen.fr; 4 Département Technologie des Polymères et Composites & Ingénierie Mécanique (TPCIM), Institut Mines Telecom Lille Douai (IMT Lille Douai), 59508 Douai, France; jeremie.soulestin@imt-lille-douai.fr

**Keywords:** biodegradable polymers, coextrusion, multilayer film, barrier properties, montmorillonite fillers

## Abstract

Biodegradable PLA/PBSA multinanolayer nanocomposites were obtained from semi-crystalline poly(butylene succinate-*co*-butylene adipate) (PBSA) nanolayers filled with nanoclays and confined against amorphous poly(lactic acid) (PLA) nanolayers in a continuous manner by applying an innovative coextrusion technology. The cloisite 30B (C30B) filler incorporation in nanolayers was considered to be an improvement of barrier properties of the multilayer films additional to the confinement effect resulting to forced assembly during the multilayer coextrusion process. 2049-layer films of ~300 µm thick were processed containing loaded PBSA nanolayers of ~200 nm, which presented certain homogeneity and were mostly continuous for the 80/20 wt% PLA/PBSA composition. The nanocomposite PBSA films (monolayer) were also processed for comparison. The presence of exfoliated and intercalated clay structure and some aggregates were observed within the PBSA nanolayers depending on the C30B content. A greater reduction of macromolecular chain segment mobility was measured due to combined effects of confinement effect and clays constraints. The absence of both polymer and clays interdiffusions was highlighted since the PLA glass transition was unchanged. Besides, a larger increase in local chain rigidification was evidenced through RAF values due to geometrical constraints initiated by close nanoclay contact without changing the crystallinity of PBSA. Tortuosity effects into the filled PBSA layers adding to confinement effects induced by PLA layers have caused a significant improvement of water barrier properties through a reduction of water permeability, water vapor solubility and water vapor diffusivity. The obtaining barrier properties were successfully correlated to microstructure, thermal properties and mobility of PBSA amorphous phase.

## 1. Introduction

Over the past thirty years, numerous works have been carried out in the field of polymer materials with the aim of reducing permeability as much as possible and approaching properties of other materials like glass or metal. The strategies developed to achieve this aim were the deposition of a thin layer by cold plasma [1,2]; the mixtures of highly barrier polymers, like EVOH for example with standard polymers used in packaging field like PE [3], PET [4] or PP [5]; the incorporation of fillers like montmorillonite [6,7] or graphene [8,9]; and even the development of a multilayer structure, containing 5–7 layers, each one having a specific characteristic. During the last 15 years, an innovative coextrusion process has been developed at the laboratory scale, allowing production of multinanolayer films containing up to 2000 or 4000 layers of two different polymers [10]. The most important results concerning the improvement of the barrier properties thanks to this multinanolayer structure, were obtained by confining PEO with EAA [11] and PS [12] or PCL with PS [13] and PMMA [14]. The confinement effect, induced by the forced assembly multinanolayer co-extrusion process, led to a spectacular decrease in the oxygen permeability coefficient of more than 20 times and was explained by the induced morphology of the crystals creating more tortuosity into the film. In fact, in multilayered structures by nanoscale confinement, the polymer layers crystallized as single crystals in in-place orientation parallel to the layers. More recently, the confinement of PLLA by PC and PS amorphous polymer layers have been performed by means of the multilayer coextrusion process [15], and the authors have highlighted the impact on the oxygen barrier properties of the rigid amorphous fraction (RAF) generated by postannealing of the multilayer films. Annealing PLLA/PS films allowed for obtaining a high crystallinity degree without RAF and this has induced an oxygen barrier improvement by a factor of 10 with 20 nm thick PLLA layers [15]. Concerning the water barrier properties for multilayer films, nowadays, only few works refer to this and the water barrier improvement was mainly obtained afterheat treatment or after a biaxial drawing [16] utilized to promote polymer crystallization. Such post treatments have improved water barrier properties. Interesting results were put forward with PC/MXD6 multilayer films [17] without annealing treatment. The authors have reported improvement of water barrier properties, only due to the nanoscale confinement effect induced by the alternating polymer multilayering, even if water-crystallization effect for MXD6 was evidenced causing an increase in MXD6 crystallinity. Again, in a previous work, a PLA/PBSA [18] multilayer film was elaborated and a reduction in permeability coefficient to different molecules was observed, by 100 times in the case of carbon dioxide. In order to further improve the barrier performances of this material, it has been considered the possibility to couple the multilayer structure with one of the approaches mentioned above, namely by incorporating fillers into the multilayer structure. Until now, very few works have taken advantage from the multinanolayer process in order to disperse and/or orientate fillers (microtalc [19], phosphate glass [20], carbon black [21], montmorillonite [22], carbon nanotubes [23], graphene [24,25]) in the aim to improve mechanical and/or barrier properties. For example, an increase of the stiffness (Young’s modulus from 2.5 GPa to 5 GPa) with the addition of 20% of phosphate glass fillers into a PP-g-MA multilayer film [20] or a mechanical reinforcement of the layers by 118% with the incorporation of 2% of graphene into PMMA multilayer structure [24] have been reported. Concerning gas barrier properties, a decrease of around 30% for the oxygen permeability coefficient has been measured with the multilayer film of PP-g-MA containing phosphate glass fillers [20] (around 20 times after bi-drawing treatment, which allows us to lengthen the fillers, changing from a spherical to a lamellar form). For a multilayer film composed of alternating layers of LDPE and LDPE-g-MA filled with 5 wt% of montmorillonite C20A [22], a reduction of the oxygen permeability coefficient from 0.84 to 0.14 Barrer was obtained after an annealing step leading to an orientation of clay particles in the plane of the layers.

In this work, PBSA nanocomposite films and PLA/PBSA (80/20 wt%) multinanolayer films loaded with organo-modified montmorillonite (C30B) were investigated. The intent of this work is to show how the incorporation of lamellar clays and their dispersion in PBSA nanolayers confined by PLA layers influence the mechanical and barrier properties, given that, to our knowledge, such a biodegradable nanomaterial has never been developed and studied in literature. The water transport properties of these nanomaterials were analyzed from permeation and sorption measurements, as well as their thermal and mechanical properties in relation with their microstructure.

## 2. Materials and Methods

### 2.1. Materials

Poly(lactic acid) (PLA), under the trade of 4060D corresponding to an amorphous polymer, was provided by Resinex (France). Semicrystalline poly(butylene succinate-*co*-butylene adipate) (PBSA), referenced as PBE001, was supplied by NaturePlast (France). Organo-modified montmorillonite Cloisite 30B (noted C30B in this study) was supplied from BYK Additives. This filler was selected owing to its better dispersion into the PBSA matrix [26] compared to native montmorillonite filler due to the organomodification.

### 2.2. Films Preparation

The incorporation of C30B clays into PBSA matrix was carried out in two steps. The first step consists in preparing a masterbatch containing 25 wt% of filler. Briefly, PBSA pellets were dried at 80 °C overnight and melt-mixed with clays by using a Coperion ZSK 26 twin-screw extruder at a temperature of 210 °C with a screw speed of 250 rpm. At the end of the die, the extrudate was cooled down in liquid water, pelletized and then dried at 80 °C to remove residual water. The second step concerns the dilution of the masterbatch with unfilled PBSA by twin-screw extrusion in order to obtain nanocomposite films with 2 wt% and 5 wt% of fillers. The same extrusion profile was used with pelletization and drying. The resulting C30B-PBSA pellets were thereafter dried before the elaboration of the nanocomposite films, as explained below.

The monolayer films of PBSA with 2% and 5 wt% of fillers were prepared using a classical single-screw extruder (20/20D Scamex extruder) with a 120/150/150/160/170 °C temperature profile and a screw speed of 29 and 33 rpm, respectively. The final thickness of films was obtained around 200 µm. Using the same parameters, the film of neat PBSA was also extruded. The films were thereafter noted as PBSA, PBSA2 and PBSA5 for the films containing 0, 2 and 5 wt% of fillers, respectively. 

Multilayer films were prepared by applying the multilayer co-extrusion process which consists of melt PLA and PBSA into two separate single-screw extruders that converge in a three-layer feedblock to form an A/B/A structure, which is then multiplied by using the multiplying elements, as detailed in a previous paper [18]. The temperature profiles were 165/180/180/190/190/190 °C and 120/150/150/160/170 °C for the PLA and the PBSA, respectively. In this study, 10-layer multiplying elements (LME) at a temperature of 170 °C were used in order to obtain a film with theoretically 2049 layers. The PLA/PBSA multilayer films were composed of 80 wt% of PLA and 20 wt% of PBSA (unfilled or filled). The resulting films were noted as PLA/PBSA, PLA/PBSA2 and PLA/PBSA5 for the multilayer films containing 0, 2 and 5 wt% of C30B into the PBSA layers, which correspond at a final filler content of 0.5% and 1.25% into the two multilayer films. Considering a final thickness of 300 µm for the multilayer film, the individual thickness of the PBSA layers should be around 60 nm in theory.

In order to better evaluate the impact of the fillers within the PLA/PBSA multilayer film, a monolayer film of PBSA was investigated. The nanocomposite films of PBSA were thereafter named reference films in the second part of the characterization of the multinanolayer films. 

### 2.3. Morphological Characterization

For Transmission Electronic Microscopy (TEM), the cross-section of the films has been prepared with an ultramicrotome (Leica UC7 cryomicrotome) at −140 °C using a diamond knife cryo immuno from Diatome (Switzerland) at a cutting speed of 1 mm/s to obtain ultrathin smooth slices of 80–90 nm thick. The films were imaged with a FEI Tecnai 12 Biotwin equipped with a thermo-ionic electron gun in LaB6 and operating at a tension of 80 KeV. TEM is equipped with CCD erlanghen ES500W camera. The different images were presented with the Digital Micrograph Software.

XRD analysis were performed on a Bruker AXS D8 Advance diffractometer with a Cobalt radiation source at a length wave of 1.789 Å. WAXS diffractograms were obtained in normal direction over the 2θ range from 5 to 40° with an angle increment fixed to 0.04° per step and a scan speed set to 1 sec/step. All scattering peaks and amorphous halos were fitted assuming Lorentzian equation.

### 2.4. Thermal Analyses

Thermal gravimetric analysis (TGA) was used in order to quantify the filler amount into the PBSA matrix. The analyses were performed at a heating rate of 10 °C/min from 30 to 550 °C under nitrogen atmosphere on a Q500 TGA from TA Instruments.

DSC Q2000 from TA instruments was used to perform DSC and MT-DSC analyses. DSC mode was used to determine the characteristic temperatures (*T_g_*, *T_c_* and *T_m_*) and the degree of crystallinity (*X_c_*) of polymers and MT-DSC mode to determine the mobile amorphous fraction (MAF) and rigid amorphous fraction (RAF). The DSC measurements were made with sample of ~6 mg at a heating/cooling rate of 10 °C/min from −60 to 200 °C, and the MT-DSC measurements were conducted in “Heat-only” mode (oscillation amplitude: 0.21 °C, oscillation period: 80 s, heating rate: 1 °C/min) with ~3 mg of polymer from −90 to 200 °C.

The degree of crystallinity was determined from the following equation:

(1) $$X_{c}\left(\%\right)=\frac{\Delta H_{m}-\Delta H_{c}}{\Delta H_{m}^{0}\;\left(1-\phi \right)}\times 100$$

where DHm is the melting enthalpy, DHc is the enthalpy of crystallization and $\Delta H_{m}^{0}$ is the melting enthalpy of a 100% theoretical crystalline polymer. For the PBSA,$\;\Delta H_{m}^{0}$ is equal to 113.4 J/g [27]. In the case of loaded PBSA, *ϕ* is the mass fraction of fillers.

The mobile amorphous fraction (*MAF*) was calculated from the glass transition by using the equation:

(2) $$X_{MAF}\left(\%\right)=\frac{\Delta C_{p}}{\Delta C_{p}^{0}}\times 100$$

where DCp is the specific heat capacity of PBSA and $\Delta C_{p}^{0}$ is the specific heat capacity for the 100% amorphous PBSA polymer. The $\Delta C_{p}^{0}$, equal to 0.614 J·g^−1^·°C^−1^, was previously determined by Bandyopadhyay et al. [28]. 

The rigid amorphous fraction (*RAF*) was obtained from the following equation:

(3) $$X_{RAF}\left(\%\right)=100-\left(X_{c}-X_{MAF}\right)$$

### 2.5. Mechanical Tests 

Uniaxial mechanical tests were performed on at least ten samples of 30 mm in length and 4 mm in width. The tests were conducted at room temperature (23 °C) on an Instron 5543 traction machine equipped with a 500 N load cell and with a speed of 25 mm/min. From the stress-strain curves, the mechanical parameters Young’s modulus, tensile strength and the elongation at break were determined.

### 2.6. Barrier Properties

Water permeation experiments were performed at 25 °C on a lab-made apparatus, as detailed in a previous paper [29]. The principle is based on the monitoring of the dew point of the permeate gas using a chilled mirror hygrometer (Elcowa, France, General Eastern Instruments). The apparatus is composed of a two-compartment measurement cell and the studied film is placed between the two compartments. A preliminary drying step by nitrogen flux is conducted to obtain a low constant dew point close to −70 °C. The measurement was started when liquid water was introduced into the upstream compartment of the measurement cell and the diffusion of water through the studied film is monitoring at downstream from the variation of the dew point temperature as a function of time. The resulting permeation flux curve allows for determining the stationary flux *Jst* (mmol·cm^−2^·s^−1^) at the stationary state as follows:

(4) $$J_{st}=\frac{f\times 10^{-6}\times \left(x^{out}-x^{in}\right)\times p_{t}}{A\times R\times T}$$

with *f* the flow rate (560 mL·min^−1^), *A* the exposed area (2.5 cm^2^), *R* the ideal gas constant (0.082 atm·cm^3^·K^−1^·mmol^−1^), *T* the temperature of the experiment (298.15 K), *p_t_* the total pressure (1 atm), *x^in^* et *x^out^* the inlet and outlet water contents (ppm) present in the sweeping. These contents were calculated with the following equation:

(5) x=e(−bTdp+c)

where *b* and *c* are empirical constants (*b*: 6185.66 K and *c*: 31.38) valid for a dew temperature range from −75 °C to −20 °C [30] and *T_dp_* the dew point temperature.

At the stationary state, where the stationary flux, *Jst*, is proportional to the permeability coefficient *P*, and considering that Δ*a* = 1, the permeability coefficient can be determined from:

(6) $$P=\frac{J_{st}\times L}{\Delta a}$$

with *Jst* the stationary flux, *L* the thickness of the film and Δ*a* the difference in water activity between the two faces of the film (in our case Δ*a* = 1). 

In terms of diffusivity, the water diffusion coefficient is calculated from the slope of the permeation curve by plotting the normalized water flux *J*/*J_st_* as a function of the reduced time t·L^−2^. In the case of vapors, the variation in diffusion with water concentration during permeation course is usually fitted by an exponential law reflecting a water concentration-dependence of the diffusion coefficient. This dependence is due to the water plasticization phenomenon inducing generally an increase in free volumes within the films. This well-known phenomenon, considered as a Fickian process of type B, conforms to:

(7) $$D=D_{0}\times e^{\left(\gamma C\right)}$$

where *D*_0_ is the diffusion coefficient when the water concentration is close to 0 at the starting of measurement, γ is the plasticization factor, and *C* is the local water concentration. The parameters were determined from the mathematical approach developed in [31]. At the stationary state, when *C* is equal to *C_eq_*, the maximum coefficient diffusion *D_m_* can be determined from:

(8) $$D_{m}=D_{0}\times e^{\left(\gamma C_{eq}\right)}$$

where *γC_eq_* is known as the plasticization coefficient. 

Water vapour sorption measurements were performed at 25 °C on a gravimetric Dynamic Vapor Sorption analyser (Surface Measurement Systems Ltd., London, UK) equipped with an electronic Cahn 200 microbalance (mass resolution of 0.1 µg). The water activity ranging from 0 to 0.95 was adjusted by mixing dry and saturated nitrogen gases using electronic mass flow controllers. The film sample placed in a pan was predried at 0% RH by exposure to dry nitrogen flux until no further change in dry weight was measured and was subsequently hydrated. For each water activity tested, the film mass was monitored as a function of time until a constant value was obtained. The correspondence between water activity and the corresponding water gain mass at equilibrium state (expressed in g of water per 100 g of polymer film) led to plotting the sorption isotherm curves. The isotherms curves for the tested films were modelled using two sorption modes, the Henry-law sorption and the aggregation (clustering) sorption, as represented in Equation (9). This combination of the two sorption modes is appropriate to fit the sorption data since the mean deviation moduli MDM calculated by the root sum square method (RSS method) are lower than 10% [32], attesting to the good fitting. The curve profiles (linear then exponential) is so characteristic of a random dissolution of water molecules in the polymer amorphous phase (Henry-law sorption), followed by an exponential increase of water mass gain related to the aggregation phenomenon of water molecules. The water concentration at an equilibrium state depending on the water activity according to the two sorption modes, Henry-law and aggregation sorptions, can be expressed as follows [33]:

(9) $$C=k_{D}\times a+n\times K_{a}\times k_{D}^{n}\times a^{n}$$

where *k_D_* (Henry’s constant) is the constant related to dissolution of permeants in the polymer matrix, *K_a_* is the equilibrium constant associated with the water aggregation reaction and *n* is the mean number of water molecules constituting the aggregates. 

From water vapor sorption kinetics, two diffusion coefficients were calculated to evidence the transfer of water molecules by random molecular motions through the films. Based on Fick model, the analytical expression of the sorption advancement *M_t_*/*M_eq_* as a function of dimensionless time *τ* (=*Dt*/*L*^2^) is:

(10) $$\frac{M_{t}}{M_{eq}}=1-\frac{8}{\pi^{2}}\overset{\infty }{\underset{n=0}{\sum }}\frac{e^{-{\left(2n+1\right)}^{2}\times \pi^{2}\times \tau }}{{\left(2n+1\right)}^{2}}$$

The resolution of Equation (10) led to the determination of two coefficients as a function of the extended time of the sorption process. In that case, the diffusion coefficient noted *D*_1_ is related to the diffusion for short time (i.e., when *M_t_*/*M_eq_* < 0.5) (Equation (11)) and the diffusion coefficient noted *D*_2_ is related to the diffusion for longer time (i.e., when *M_t_*/*M_eq_* > 0.5) (Equation (12)).

(11) $$\frac{M_{t}}{M_{eq}}\approx \frac{4}{L}\sqrt{\frac{D_{1}}{\pi }}\sqrt{t}$$

(12) $$\ln(1-\frac{M_{t}}{M_{eq}})\approx \frac{-\pi^{2}\times D_{2}}{L^{2}}\times t-\ln\left(\frac{\pi^{2}}{8}\right)$$

## 3. Results and Discussion

### 3.1. Impact of Montmorillonite C30B Fillers on the PBSA Matrix

#### 3.1.1. Microstructure Examination by Microscopy and WAXS

**Figure 1:**
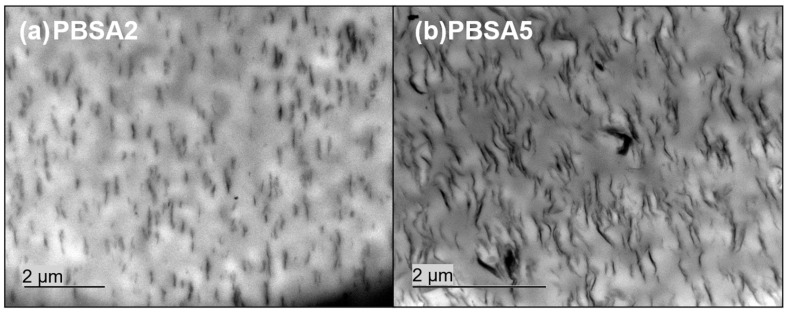
TEM observations for PBSA filled with (**a**) 2% and (**b**) 5 wt% of montmorillonite C30B.

In order to determine the state of dispersion of montmorillonite C30B fillers into the PBSA matrix, observations by transmission electron microscopy (TEM) were done. The obtained images are gathered in Figure 1. The dispersion of 2 wt% of C30B fillers into PBSA seems to be homogeneous with only very small aggregates in some places. As expected, when the filler content increased up to 5 wt%, the C30B fillers were less individualized and the number of aggregates appeared to be more important. In both cases, the orientation of C30B fillers specifically in the extrusion flow direction is obviously confirmed. The PBSA based films filled with C30B can be considered as nanocomposites with a coexistence of intercalated and exfoliated inorganic structures. 

**Figure 2:**
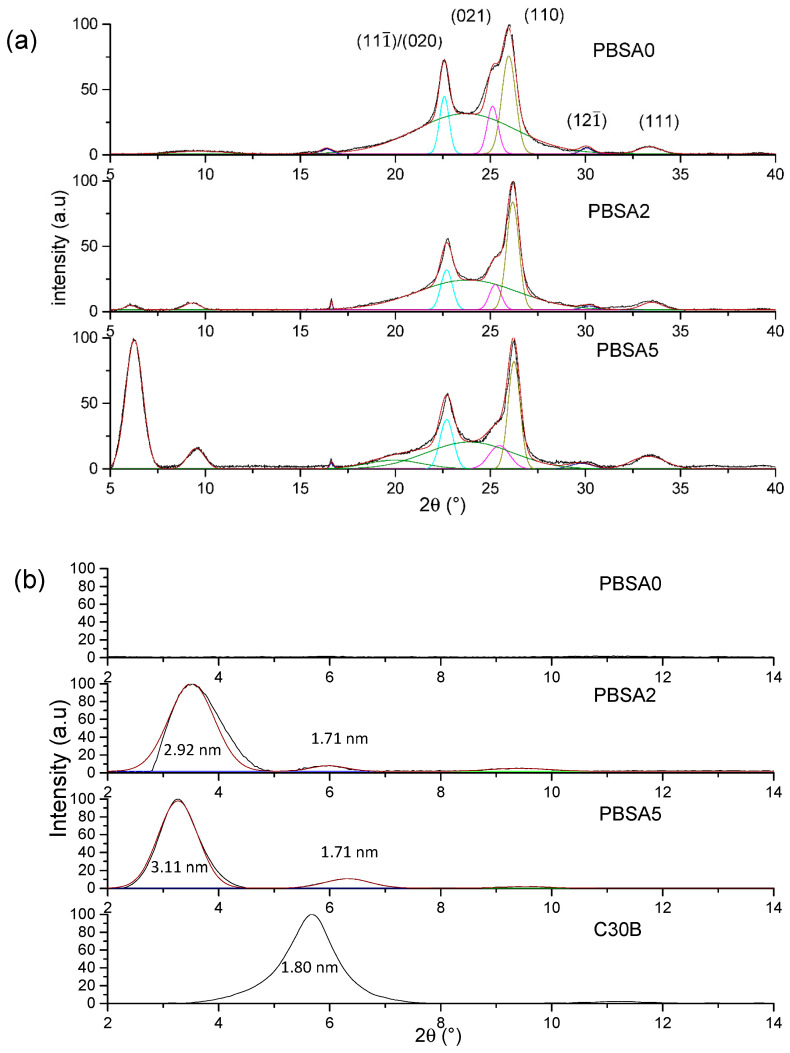
XRD spectra of neat PBSA film and filled PBSA films (**a**) in the 15–40° range and (**b**) in the 2–14° range.

To complete these observations, WAXS measurements were conducted on the neat and the nanocomposite PBSA films to evaluate the structure and microstructure of films. The DRX diffractograms for the films are presented as a function of 2θ range in Figure 2.

First, PBSA being a semicrystalline polymer, the crystalline structure is evaluated specifically in the 15–40° range (Figure 2a) in agreement with the appearance of mean diffraction peaks relative to polymer crystals. Crystallizing in monoclinic crystal lattice [34], the four strong diffraction peaks are located at 2θ = 22.6°, 25.2°, 26.0°, 30.1° and 33.4° relative to (11$\bar{1}$)/(020), (021), (110), (12$\bar{1}$) and (111) diffraction planes, respectively, as previously reported [35] with the amorphous halo centered on 2θ = 24°. The diffractograms of the filled PBSA films can be superposed to the neat PBSA one, meaning that the filler incorporation has any impact on the crystalline phase of PBSA. A small difference in terms of peak intensity is only noted due likely to a certain anisotropy of crystals within the films. 

In the diffraction angle 2–15° range, the dispersion and exfoliation level of fillers in the PBSA is evaluated (Figure 2b) in accordance with the diffraction patterns of nanoclays. The interlayer distances (inserted in Figure 2b) relative to diffraction peaks were calculated by using the Bragg law. As indicated in the literature and reported in this work, the interlayer distance of C30B is equal to 1.80 nm. For the filled PBSA films, the diffraction peaks detected at 3.5° and 3.3° for 2% and 5% of C30B, respectively, indicates exfoliation and intercalation of nanoclays in PBSA matrix. The interlayer distance is found to be higher than that of the C30B powder and increases with the filler content, attesting an improvement of dispersion and exfoliation levels. Indeed, the peak at 2θ = 6.0° was assigned to a second intercalated structure with lower interlayer space (1.71 nm) arising from the degradation of C30B surfactant [36] and/or platelet re-aggregation by clay contact. As a result, this is larger with the highest filler content. This result agrees with the TEM observations. One can conclude that C30B nanoclays are well dispersed and exfoliated within the PBSA matrix with an increase in interlayer distances as filler content increases, even if one can observe aggregates from TEM observations. 

#### 3.1.2. Thermal and Mechanical Analyses 

**Figure 3:**
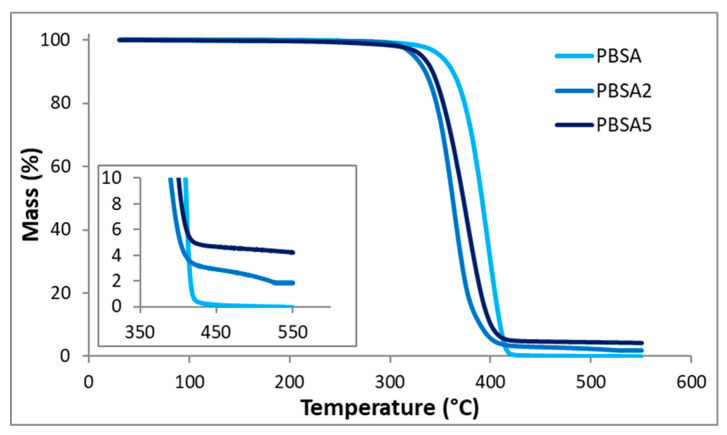
TGA curves obtained for the neat PBSA film and the filled PBSA films.

A thermogravimetric analysis was performed on the PBSA films filled with montmorillonite C30B fillers to quantify the exact proportion of fillers incorporated in PBSA. The value fits the residual percentage after total degradation of polymer at high temperature. The TGA curves reported in Figure 3 confirm the filler content of the PBSA2 and PBSA5 films.

Then, considering the TGA profiles, it is worth noting that the degradation temperature of the PBSA (determined at 5% of mass loss) decreased with the filler content. Indeed, the degradation temperature of 350 °C for the neat PBSA film was reduced to approximately 330 °C for the nanocomposite films. To explain this phenomenon, two hypotheses can be given: the first one concerns the presence of surfactant included in the organo-modified montmorillonite that has induced a degradation temperature of 174 °C for C30B [37], lower than that of the PBSA polymer and reducing the degradation temperatures of the filled-PBSA films. The second one is linked with the fact that two steps were necessary to obtain the loaded films, inducing longer residence times in the extruder that may cause of a reduction in size of macromolecular chains, leading to a faster degradation compared to neat PBSA.

DSC and MT-DSC analyses were performed in order to determine the characteristic temperatures (*T_g_*, *T_c_* and *T_m_*), the degree of crystallinity (*X_c_*) and the specific heat capacity Δ*C_p_*, to quantify the mobile amorphous fraction (MAF) and the rigid amorphous fraction (RAF) for the films using Equations (2) and (3). The results are gathered in Table 1. It is worth noting that similar crystallization and melting temperatures are measured before and after incorporation of the fillers and that the crystallinity degree remains unchanged (~38%). However, a slight increase of the glass transition temperature is measured, meaning the macromolecular chains involved in the amorphous phase of PBSA are less mobile, probably due to constraints exerted by the nanofillers.

Considering that the incorporation of fillers into the PBSA matrix did not affect the crystallization, it may be of interest to evaluate the effect of the fillers on the amorphous phase through the change in Δ*C_p_* calculated at the glass transition. Indeed, the heat capacity step normalized to the polymer fraction is directly linked to the mobile amorphous fraction MAF according to Equation (2). A decrease of Δ*C_p_* value would mean that there is less mobile amorphous fraction (MAF) and consequently more rigid amorphous fraction (RAF) in the filled films. The presence of the C30B fillers in PBSA led to increase the percent of RAF by a factor two (Table 1). Such an increase in RAF by incorporation of fillers has already been observed in PBSA [38], in PU [39] or in an hyperbranched polyester [40] for example. This significant decrease of Δ*C_p_* values, evidencing an increase in local rigidity, can be due to the reduction in free volumes in polymer by addition of fillers and the resulting mobility restrictions of macromolecular chains coming from the presence of fillers. From our results, we can infer that the C30B fillers dispersed in the PBSA matrix cause geometrical constraints to amorphous polymer chains, resulting in the increase of the glass transition temperature and the increase of the RAF.

Mechanical analyses were performed and the data are gathered in Table 2. The values obtained for the neat and filled PBSA films are convenient with values presented in the literature [18,38,41,42]. The high values of elongation at break for films reveal the ductile behaviour of PBSA and are in the same order of magnitude for the neat and filled films [18]. It is worth noting an increase of the Young’s modulus and a decrease of the yield stress and the elongation at break when PBSA is filled with montmorillonite. This trend is classically observed with nanocomposites [43] and reported in the case of films of PLA [44], PBS [45,46], PBSA [47] or PU [48]. A high increase in Young’s modulus (in our case around 50% for 5 wt% of C30B) is usually correlated with a good exfoliation and dispersion of fillers within the polymeric matrix. The result is thus an indirect way to evidence the good dispersion and exfoliation of C30B within PBSA. This increase of stiffness could be associated with the RAF increase. 

#### 3.1.3. Water Barrier Properties 

**Figure 4:**
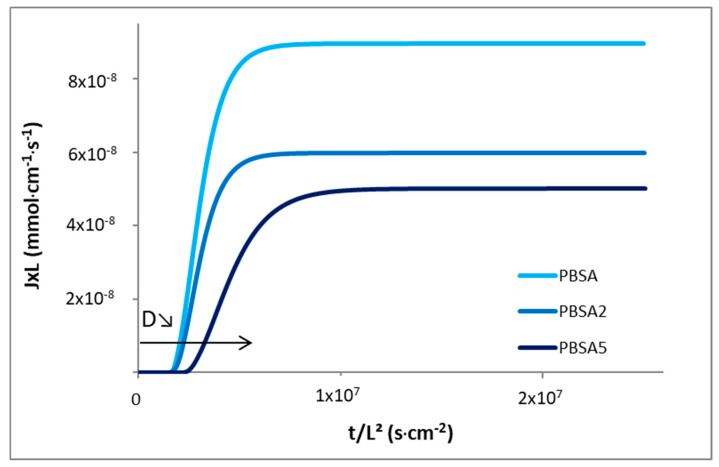
Reduced water permeation curves for the neat PBSA and the filled PBSA films.

The permeation kinetic curves of the neat and filled PBSA films are plotted in Figure 4 in the reduced scale J*L = *f*(*t*/*L*^2^) in order to overcome film thickness effects. The water permeation flux is found to be faster for the PBSA film until a higher constant value at the steady state is measured. As a result, the permeability coefficient was found to higher than the values of the filled films. Considering the steady state of water permeation, the permeation flux shifted to lower values with the increase in filler content, indicating a reduction of the water diffusivity through the nanocomposite films. These results clearly attest for an increase of water barrier properties by incorporation of impermeable fillers within the semicrystalline polyester. This barrier effect is usually attributed to the tortuosity effect induced by the presence of nanoclays, but could also be correlated, in part, to the increase of RAF for the nanocomposite films (9% vs. 18%, Table 1). The mobility of the amorphous chains at the vicinity of crystals was locally reduced by geometrical constraints which hinder passage of water molecules. A shift of the reduced permeation flux curves towards longer time is so obtained, highlighting a delay time in diffusion, which suggests an increase in the diffusion pathways with the filler incorporation. These observations are in line with the decrease of diffusion coefficients *D*_0_ and *D_M_* determined from Equations (7) and (8). The incorporation of C30B within PBSA has thus induced a decrease of permeability and diffusion coefficients, as shown in Table 3. As thoroughly reviewed by Tan and Thomas [49], one can explain these results by a strong improvement of water barrier effect due to tortuosity induced by the dispersion and exfoliation levels of lamellar fillers within the polymer matrix. In such a case, permeability and diffusion coefficients are accordingly reduced, as obtained in this work.

As commonly observed with water as diffusing molecules for polyester films [18,35,50], a plasticization effect of the polymer by sorbed water is also obtained. The water diffusion coefficients are accordingly dependent to the sorbed water concentration and this dependence is usually described through the exponential law *D = D*_0_*.e^γC^* [51]. The experimental water permeation fluxes of films were successfully fitted and the calculated parameters are gathered in Table 3. The water-induced plasticization is clearly observed since *D*_0_ (diffusion coefficient at water concentration close to 0) is found to be lower than *D_m_* (diffusion coefficient at water concentration at equilibrium state) for the three films (Table 3). The difference between *D*_0_ and *D_m_* values is smaller for the nanocomposite films with the increase of filler content. This result highlights a reduction of plasticization effect with the filler incorporation, which is correlated with the reduction of the plasticization coefficient *γC_eq_*, even if the water concentration at equilibrium state is rather constant (~0.7–0.8 mmol·cm^−3^). It seems that the plasticization ability of PBSA which leads to increase the free volumes is reduced. This behaviour could be due to the reduction in macromolecular chain segment mobility, as shown from the increase of stiffness and by RAF increase, but without changing the sorbed water content. 

**Figure 5:**
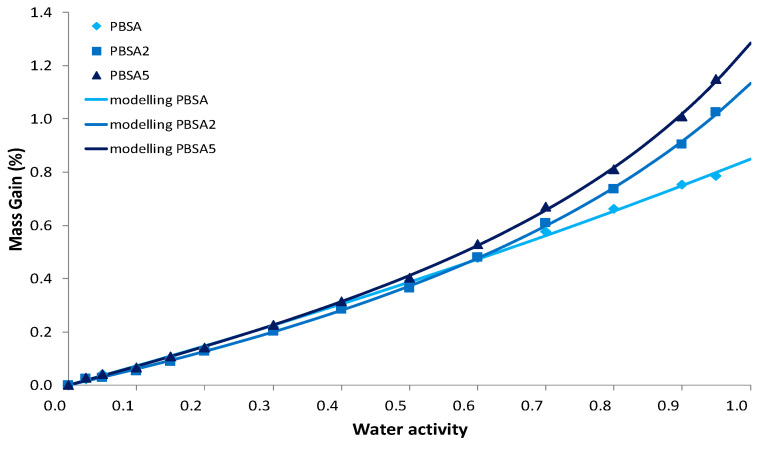
Water permeation curves for the neat PBSA and the filled PBSA films fitted using a model combining two sorption modes, the Henry-law sorption and the aggregation (clustering) sorption, as represented in Equation (9).

In the case of the water vapor sorption phenomenon, the sorbed water concentration in films was measured as a function of time for water activity range from 0 to 0.95. The size and the thickness of the film samples are unchanged after sorption measurements. The corresponding water vapor sorption isotherms (equilibrium state of sorption kinetics) for the neat and the filled PBSA films are plotted in Figure 5. The shape of the isotherm curves is rather similar whatever the tested film: a linear increase of water mass gain as water activity *a_w_* increases, followed by a sharp rise of water mass gain at *a_w_* > 0.6. As largely reported in the literature [52,53,54], C30B filler sorb a higher water content than polyester or polyamide films within the whole water activity range, because of water-sensitive groups, such as silicate and hydroxyethylene groups, included in the surfactant of C30B [55]. From Figure 5, the water vapor sorption behaviour of the filled PBSA films deviates from that of the neat PBSA film at *a_w_* > 0.5. The C30B incorporation in PBSA clearly contributes to this deviation in water solubility from *a_w_* = 0.5, testifying to the hydrophilic nature of clays, even if there is an organomodification. This deviation in water mass gain is greater with the filler content increase, in correlation with their hydrophilic nature. It is worth noting that the increase in water solubility shows an opposite trend compared to the reduction of water permeability coefficient obtained by permeation measurement. One can specify here that the permeability coefficient results of two contributions, one is the solubility and the other the diffusivity. For this latter contribution, the reduction of water diffusivity is expected by increasing of tortuous diffusing pathways owing to the presence of impermeable fillers in the PBSA matrix. In addition, in the case of sorption process, the increase in water solubility by increase in water mass gain is mainly due to the hydrophilic nature of C30B in comparison with the sorption capacity of PBSA polymer. In terms of water transport properties, one can infer that C30B filler is responsible for an increase of tortuosity relative to the film microstructure and filler organisation, and for an increase in solubility relative to the presence of water-sensitive groups. 

Regarding isotherm curves, such a curve profile can be appropriately fitted using Equation (9) which refers to the combination of Henry-law and aggregation sorption modes, as shown by the fitted curves reported in Figure 5 as well as by the mean deviation modulus MDM lower than 10% (Table 4), both attesting to the convenient modelling of sorption data. The linear increase of water mass gain as water activity *a_w_* increases is consistent with the random dissolution of water molecules in the amorphous phase of the polymer. At *a_w_* > 0.6, the exponential increase in water mass gain conforms to cluster formation of water molecules. The modelling parameters are summarized in Table 4. The parameters analysis points out some effect from filler incorporation. The *k_D_* parameter is slightly higher for the highest filled PBSA film: the more the water solubility in films, the more the *k_D_* constant. Although a larger number of aggregates appears at this filler content, this observation reflects the effect of larger nanoclay-polymer interfaces, which can facilitate the random dissolution of water molecules in the nanocomposite films by modifying the number and the size of micro-voids inherent of PBSA polymer. The values of *K_a_* and *n* indicate the formation of water aggregates at high water activities (*a_w_* > 0.6). Both values increase as filler content increases, confirming an increase of interactions with sorbed water molecules to initiate a multilayer water sorption. Water aggregates are formed on polar sorption sites existing in the films. However, the increase of these two parameters is not so high (Table 4) indicating that the increase of free volumes by water plasticization is limited owing to a certain rigidity of polymer chains in the vicinity of C30B fillers, but also to good compatibility between organo-modified clays and PBSA chains. 

**Figure 6:**
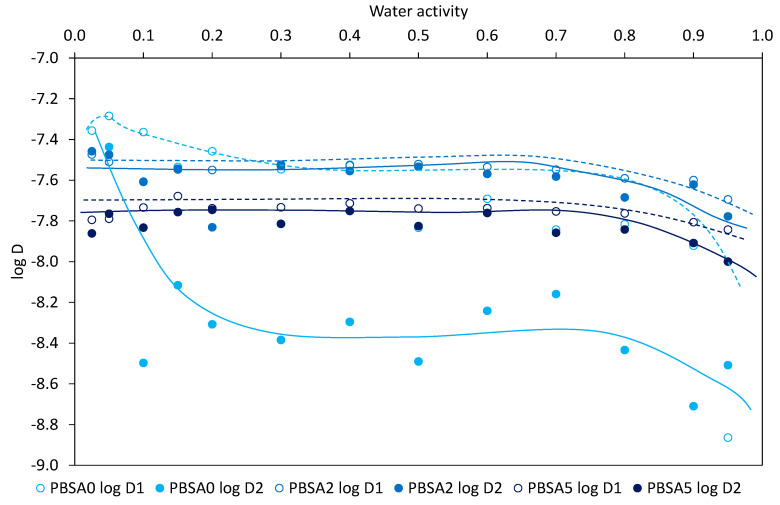
Water vapor diffusion coefficients *D*_1_ and *D*_2_ for the neat PBSA and the filled PBSA films.

The water diffusion coefficients in the neat and the filled PBSA films for the first-half sorption, noted *D*_1_, and for the second-half sorption, noted *D*_2_, are plotted in a semi-logarithmic scale (log *D*) as a function of water activity in Figure 6. It appears that the evolution of the diffusion coefficients was similar and in a same range of values whatever the film, except for the neat PBSA film where the evolution of *D*_2_ coefficient is strongly decreasing with water activity. It seems that PBSA is differently impacted by the water sorption phenomenon likely due to the plasticization effect of water vapor. It is surprising that the *D*_2_ coefficients (at longer extent of water sorption, higher water concentration) are lower than the *D*_1_ coefficients in the activity range for all films, whereas the inverse trend is commonly reported [56] due to water plasticization effects. However, such a trend has been recently mentioned in the literature for PBSA-based nanocomposites [53]. The authors outline two antagonist effects occurring for longer time that would balance each to give similar coefficients. The former increasing water diffusion is related to plasticization effects of water and the latter decreasing the water diffusion is related to the formation of water aggregates which restricts their mobility and as a result their diffusivity within the films. The general curve profiles for the filled PBSA films show a linear part and a reduction of diffusion at higher water activities, meaning that diffusion coefficients are rather constant and are then reduced by water clustering phenomenon linked to the increase in water cluster size which makes water molecules less mobile [57]. In addition, the evolution of the diffusion coefficients is in good agreement with the BET III sorption mechanism. 

For the whole water activity range, it is observed that the diffusion coefficients, *D*_1_ and *D*_2_, are lower for the filled PBSA films, highlighting the impact of fillers to the diffusion of water vapor in the PBSA films by tortuosity effects. However, this decrease in diffusion is inversely correlated with the filler content. In fact, the *D*_1_ coefficient is found to be reduced as the filler content increases while the *D*_2_ coefficient is found to be increased for the PBSA2 film and then slightly reduced for the PBSA5 film. *D*_2_ coefficient being considered representative of the water diffusion in the core of film and *D*_1_ coefficient representative of diffusion from the film surface, the latter result can be related to the filler presence in PBSA which prevents the water diffusion by tortuosity at the film surface, as observed with the water diffusion by permeation measurement. And, in addition, this can be the result of the RAF increase in the filled films generating geometrical constraints and restriction in mobility of macromolecular chains. When water molecules have penetrated within the films, the plasticization effect of sorbed water molecules occurs accompanied by an easier formation of water aggregates and by the creation of preferential diffusion pathways at the filler-polymer interfacial areas due to the hydrophilic character of the clays which both can help the diffusion of water in the core of the films. This effect is slowed down at the highest filler content due to larger water cluster sizes, as calculated by the modeling approach, and to larger tortuosity effects which both resulted in a reduction of the water cluster mobility. In all cases, the *D*_2_ coefficient is found lower than the *D*_1_ coefficient for the whole range of water activity, in a lesser extent for the filled PBSA nanocomposite films. This can be related to the filler incorporation in PBSA since acting as obstacles to diffusion which limits the water plasticization effect in the film, and hence diffusion in the film. 

For this first part of the work, one can infer that the incorporation of C30B fillers within the PBSA matrix has induced a slight increase in glass transition temperature associated with an increase in RAF for a constant degree of crystallinity, and an increase in Young’s modulus because of good filler exfoliation and dispersion level, even if some filler aggregates appear in some places. The C30B fillers are also seen as oriented in the extrusion direction of the film. From these results, the increase in stiffness of macromolecular chains and the presence of larger nanoclay-polymer interfaces have caused reduction of free volumes and restrictions in mobility of macromolecular chains by geometrical constraints by close contact with fillers. The water permeability and diffusion coefficients have been accordingly reduced. The water permeation flux is shifted to longer time evidencing an increase of tortuosity due to oriented lamellar structure of C30B in PBSA matrix. The reduction of water diffusion and water sorption phenomena are in good agreement with the morphology and the thermal and mechanical properties displayed by the films. The water barrier properties were improved with an increased stiffness of the nanocomposite films. 

### 3.2. Elaboration of PLA/PBSA Multilayer Film with Montmorillonite C30B

#### 3.2.1. Morphological Characterization and Nanofiller Dispersion

**Figure 7:**
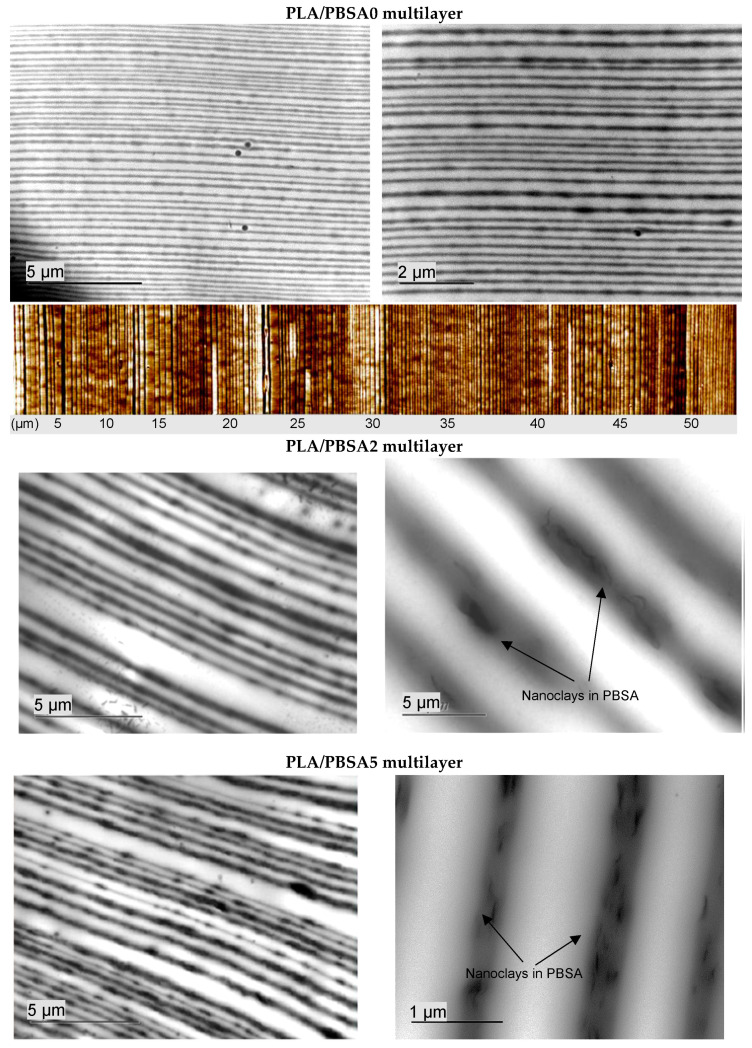
TEM observations of the unfilled PLA/PBSA and filled PLA/PBSA2 and PLA/PBSA5 multilayer films (PLA in white, PBSA in dark). (AFM image of unfilled PLA/PBSA multilayer films).

The structure of the multilayer PLA/PBSA (80/20) film was described using AFM observation in our previous work [18] and shown in Figure 7. TEM observation was considered to evidence the clay morphology and its location into the multilayer structure, as well as the multilayer structure itself. A selection of TEM images characteristics of the loaded multilayer films is gathered in Figure 7. The thickness of the PBSA layers varies from 50 to 70 nm in the neat multilayer film, as expected by calculation resulting from the number of of layers and the final thickness of the co-extruded film. A wider thickness is found for the filled PBSA layers in loaded multilayer films compared to the neat PBSA layers in the PLA/PBSA multilayer film: around 100–200 nm and 150–250 nm for the PLA/PBSA2 and PLA/PBSA5 multilayer films, respectively. The integrity of ultrathin layers of PBSA is more difficult to preserve when they are filled with clays. Some layer breakups due to clay aggregates have been observed. Nevertheless, rather homogeneous and continuous PBSA layers (black layers in the micrographs) within the multilayer PLA/PBSA structure are overall observed. One can note the presence of some filler aggregates in the PBSA layers. 

**Figure 8:**
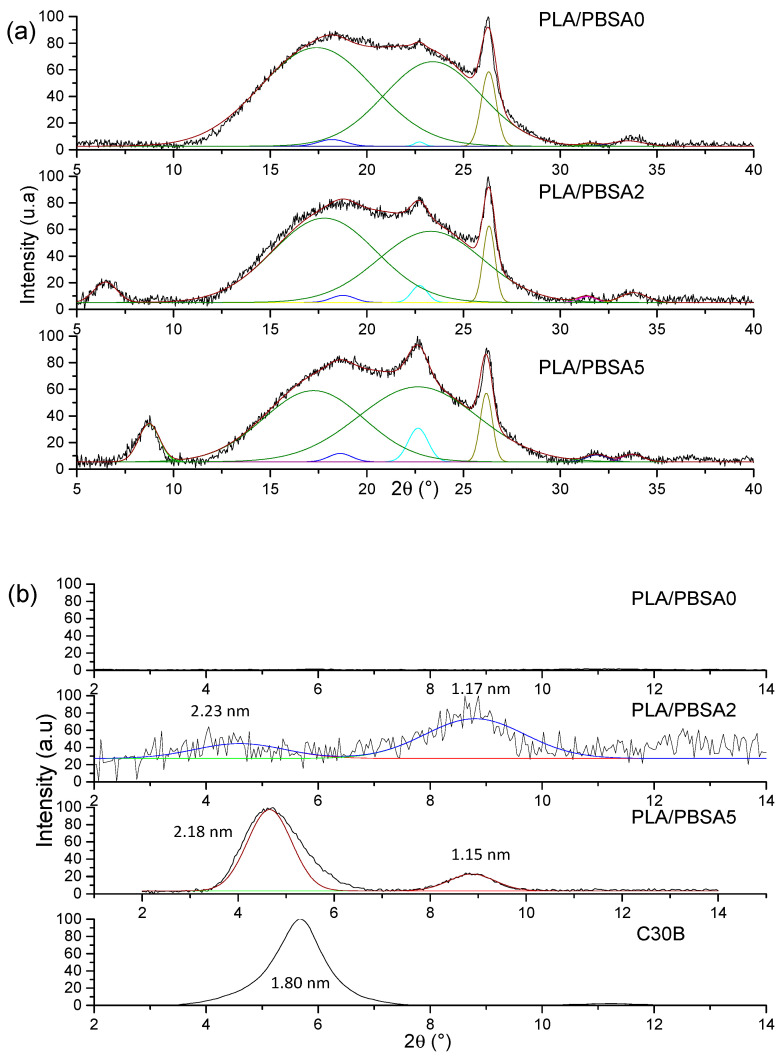
XRD spectra for the PLA/PBSA, PLA/PBSA2 and PLA/PBSA5 multilayer films in the 5–40° range (**a**) and for C30B powder in 2–14° range (**b**).

As a complementary experiment, XRD measurements were conducted on the different multilayer films to evaluate the microstructure of films. The XRD diffractograms for the multilayer films are presented in Figure 8. Recently, the diffractogram of the PLA/PBSA has been simulated from the diffractograms of each pure polymer considering the composition weight ratio [18]. The authors have pointed out a good agreement between the experimental and simulated XRD profiles, reflecting a similarity of structures for the two polymers in monolayer and multilayer organisation, especially the crystalline organisation. This result in line with the present work with a similarity in degree of crystallinity between PBSA in multilayer film (40%) and PBSA in monolayer film (38%). The PLA/PBSA0 film exhibits an amorphous halo centered on 20° relative to amorphous phase of PLA and PBSA layers and crystalline diffraction peaks relative to PBSA layers (Figure 8a). The characteristic strong peaks of PBSA at 18.2°, 22.6°, 26.2°, 31.5° and 33.5° attributed to the (11$\bar{1}$)/(020), (021), (110), (12$\bar{1}$) and (111) planes, respectively, are found by extraction of the crystalline contribution from the XRD profile. These two contributions are observed for the filled-PBSA layers multilayer films. 

In the 2–14° range (Figure 8b), the characteristic peaks for the filler were detected at 2θ = 4.7° and 8.9°. One can note an increase of the interlayer distance corresponding to an intercalated structure within the PBSA layers of the PLA/PBSA multilayer structure compared to the value of C30B powder. Indeed, the peak at 2θ = 8.9° assigned to a second intercalated structure with lower interlayer space is also visible. The relative lower amplitude of the two diffractions peaks for the PLA/PBSA2 film compared to the PLA/PBSA5 film is an indication of a better exfoliation of C30B within the PBSA layers. This observation can be explained by the difference in thickness of PBSA layers and by some filler aggregates which seem to be more present in PLA/PBSA5 multilayer film. 

#### 3.2.2. Thermal and Mechanical Analyses 

The characteristic temperatures for the multilayer films were measured by using MT-DSC measurements to well separate the PBSA crystallization observed in the non-reversible heat flow signal from the PLA glass transition phenomenon observed in the reversible heat flow signal because both events are present in the same temperature range. The values are gathered in Table 5. The thermal events are calculated from the first heat cycle to ensure that the films are in the same thermal and structural states as for permeation and sorption analyses. Those MT-DSC thermograms are a practical way to compare the thermal properties of the PLA/PBSA multilayer films as filler content increases compared to the neat PLA/PBSA multilayer film. 

It appears a significant increase of the glass transition temperature of the PBSA up to 10 °C deviation as filler content increases (Table 5). In comparison with the reference filled PBSA films (Table 1), this deviation is greater for the multilayer films (10 °C vs. 4 °C), highlighting a greater reduction of the macromolecular chain mobility. This result suggests that the constraining effect due to the clays is more pronounced in a thinner layer (100 nm on average vs. 300 µm for the reference PBSA film). As a consequence, the macromolecular chains of PBSA exhibit less mobility, requiring more energy to switch from glass state to rubbery state, which causes a higher glass transition temperature. The other characteristic temperatures are quite similar to those of the reference films. The glass transition temperature of PLA is unchanged, meaning that the clays are well contained in the PBSA layers or located at the polymer interfaces without effect on PLA chain mobility. 

For the neat and loaded multilayers films, the degree of crystallinity of PBSA in the multilayer films was evaluated (Table 5). It is worth noting a decrease of the crystallinity of PBSA with clays when the PBSA layers contains 2% of fillers. This trend is opposed to that displayed by the reference filled PBSA films (i.e., in monolayer structure) where the clays incorporation did not impact the degree of crystallinity. Such an opposite trend could be the result of confinement effect of the PBSA layers by reduction of the layer thickness generated during the forced assembly coextrusion process. The multiplication of layers by using the multiplying element device during coextrusion has drastically reduced the layer thickness (100 nm on average vs. 300 µm), restricting as a result the space available to macromolecular chains of PBSA to be properly realigned to form crystals, and also significantly increasing the local rigidity of macromolecular chains by close contact with clays in a thinner space. This is particularly noticeable with the multilayer PLA/PBSA2 film (layer thickness of 100 nm on average), for which the crystallinity is equal to 34%. In that case, the RAF is found to be of 33%, higher than the RAF of the neat PLA/PBSA, confirming the local rigidification of macromolecular chains by close clay contact in a thinner thickness. For the film with higher clay content (PLA/PBSA5), one can note an increase of the degree of crystallinity and a decrease of RAF that are similar to the unloaded PLA/PBSA multilayer. This could be attributed to a greater ease to form crystalline phase owing to a larger mobility of macromolecular chains in a thicker PBSA layer thickness (increase of 100 nm). 

Mechanical analyses (Table 6) were also performed to evaluate the impact of the clays in the PBSA layers within the multilayer structure on the mechanical performances. As the multilayer film is mainly composed of PLA (around 80 wt%), the final filler content is extremely low (0.5% and 1.25% for the two films). Therefore, no significant variations were observed. Despite a rather high standard deviation, one can only note for PLA/PBSA2 a slight decrease in the Young’s modulus and strength at break as well as an increase of the elongation at break, which could be correlated with the slight decrease of crystallinity measured by DSC.

#### 3.2.3. Water Barrier Properties 

**Figure 9:**
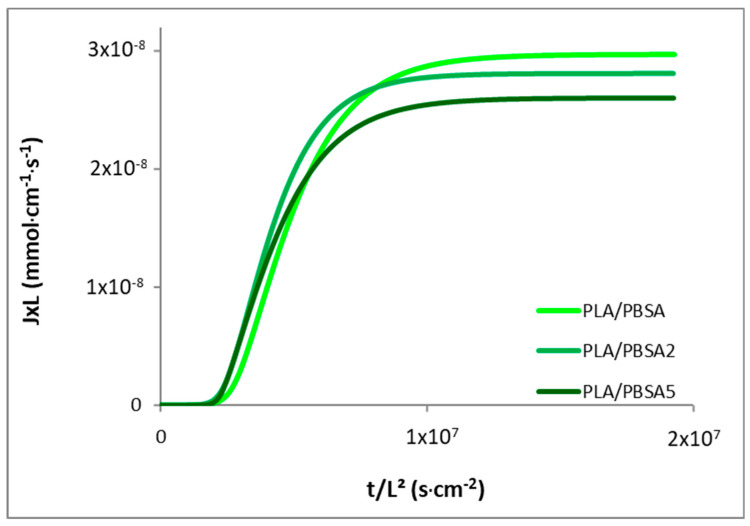
Reduced water permeation curves for the PLA/PBSA multilayer film and the filled PLA/PBSA multilayer films.

Water permeation measurements were performed on the multilayer films and the resulting water flux curves are represented in Figure 9 in a reduced scale *J* × *L *=* f*(*t*/*L*^2^) to overcome the film thickness effect. Regarding the steady state of the permeation, the water flux is shifted to lower values with filler content increases, indicating a reduction in permeability coefficients, as reported in Table 7. This improved water barrier effect is surprisingly not so high as it was expected from the oriented nanoclays more or less dispersed in nanolayers of PBSA. It must however remind that only 1.25% of C30B clays were incorporated in the multilayer film. If the water flux is reduced with the presence of fillers, a shift of the reduced water flux curves to lower time is obtained, reflecting a faster diffusion of water molecules through the filled multilayer films, as shown by the increase of diffusion coefficients *D*_0_ (Table 7). With the tortuosity effect induced by lamellar structures and the stiffness and RAF increase, it could be expected that the diffusion coefficients were accordingly reduced. This is not the case. It seems that the presence of layer breaks and of thicker PBSA layers in the filled multilayer structure has impacted the water diffusivity, regardless of the fact that C30B fillers are assumed to be impermeable entities. One can infer that the presence of very few defects in the microstructure are at the origin of a loss of macroscopic properties. Nevertheless, this increase in diffusion can be considered as low if we note the small deviation between values in comparison with the diffusion coefficient of the neat PLA/PBSA multilayer film. The low filler fraction of C30B (0.25 and 1.25 wt%) in multilayer films are probably not sufficient to highly increase the tortuous diffusion pathway of water molecules.

Again, it is worth noting that diffusion coefficient D is not constant with permeation time since *D*_0_ < *D_M_*, confirming the water plasticization effect. Even in multilayer form, the water diffusion coefficients are dependent to the sorbed water concentration. The experimental water fluxes for the multilayer films were fitted by applying the exponential law (Equation (7)) and the calculated parameters are summarized in Table 7. The deviation of *D*_0_ and *D_M_* between the values of the unfilled multilayer film with those of the filled multilayer films again confirms the faster diffusion of water in the filled multilayer films. Also, this is correlated with the filler content increase. Regarding the plasticization coefficient and the water concentration at equilibrium state, the values are quite similar, that clearly shows no real influence of the low clay fraction on the water plasticization effect. Nevertheless, in comparison with the parameters of the reference films (Table 3), it can be evidenced a large decrease in permeability and diffusion coefficients and a reduction of plasticization coefficient for the multilayer films. This effect is maintained even in presence of layer breaks in the multilayer structure. One can infer that the multilayer structure by multiplying the number of polymer layers, and hence interfaces between a semicrystalline polymer and an amorphous polymer, is at the origin of the large reduction in water transfer and its diffusion. The increase in RAF for the multilayer PLA/PBSA2 film does not seem to have impacted the barrier properties more than that. 

Considering the opposite variation of permeability and diffusion coefficients, it seems that the solubility is a key parameter in the improvement of barrier properties to water for the multilayer films where the semicrystalline polymer layers are filled with C30B. The contribution of solubility has been investigated through water vapor sorption measurements. 

By applying the series model equation as follows:

(13) $$\frac{L}{P}=\frac{L_{PLA}}{P_{PLA}}+\frac{L_{PBSA}}{P_{PBSA}}\;or\;\frac{1}{P}=\frac{\emptyset_{PLA}}{P_{PLA}}+\frac{\emptyset_{PBSA}}{P_{PBSA}}$$

where *L*, *P*, *φ* are the thickness, the permeability and the volume fraction of PLA or PBSA, respectively.

**Figure 10:**
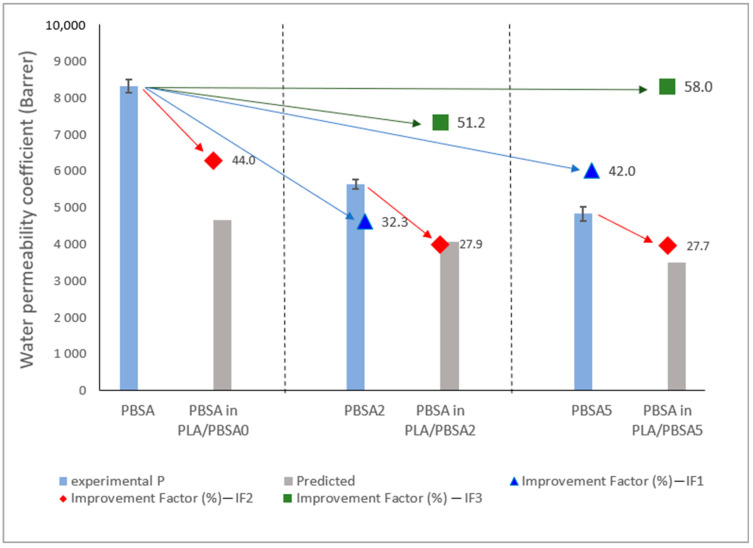
Comparison of the experimental and predicted permeabilities of PBSA under monolayer and multilayer films. ▲ IF1: calculated from neat PBSA—effect of loading ♦ IF2: calculated from PBSA (neat of filled)—effect of multilayer ■ IF3: calculated from neat PBSA—effect of loading and multilayer.

The permeability coefficient of the neat or filled PLA/PBSA multilayer films was calculated. In case of filled PBSA, the experimental permeability coefficients of PBSA2 and PBSA5 were taken from Table 3. Doing so, we can see that the experimental values of water permeability of the neat and filled multilayers are slightly lower than the calculated ones (Table 7). This result seems to show a slight barrier effect induced by the multinanolayer structure. Assuming that the water permeability of PLA is unchanged before and after loading due to its amorphous state and containing no fillers, it was then interesting to deduce the predicted water permeability coefficients of the PBSA layers in multilayer structures from Equation (13) and to see how both the nanostratification and the presence of fillers improve the water resistance of PBSA nanolayers. Results are gathered in Table 7 and are represented in Figure 10 with the experimental permeability coefficients of reference films for the sake of comparison. 

First, one can point out that the predicted permeability coefficients of PBSA layers in neat and filled multilayer films deduced from Equation (13) are lower than those of neat and loaded PBSA monolayer films. As can be speculated from the improvement factor IF (%) calculated from the difference between experimental and predicted values ((*P_exp_* − *P_predicted_*)/*P_ex_*), it was possible to highlight the effect of the presence of clays and their confinement in nanolayers on the barrier effect (Figure 10). Indeed, first by comparing IF1 and IF2, which correspond to improvement factors for the loading effect and for the multinanolayer effect, respectively, we can see that the highest values for the nanocomposite and the multilayer are quite similar, 42 and 44%, respectively. Also, when the PBSA is filled, the gain in barrier effect due to the nanostratification is ~30% and comparable to the gain when 2% of fillers are incorporated in the PBSA monolayer. In other words, it seems that the improvement of water barrier by incorporation of fillers in PBSA is similar to the PBSA layers confined by PLA layers. Consequently, it can be expected a high increase in the water barrier effect in PBSA with the presence of fillers and when confined in very thin layers. Indeed, as revealed by IF3, improvement factor that corresponds to cumulative effect of both loading and nanolayering effects, the highest improvement factor (58%) is obtained for PBSA in PLA/PBSA5.

**Figure 11:**
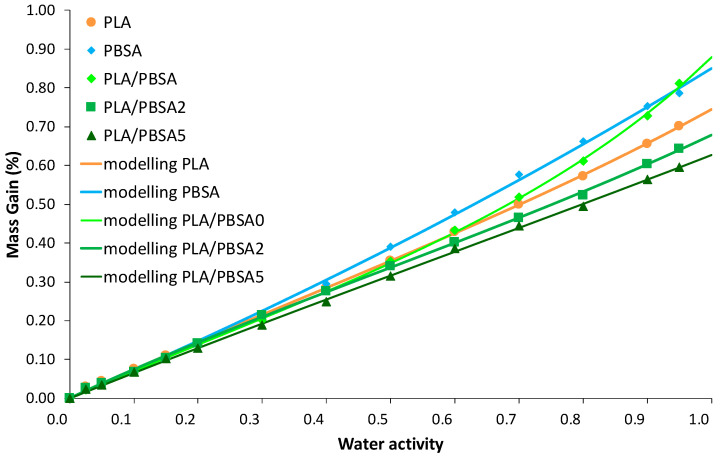
Water vapor sorption isotherms for the PLA/PBSA, PLA/PBSA2 and PLA/PBSA5 multilayer films modelled by two consecutive sorption modes, the Henry-law sorption and the aggregation (clustering) sorption, as represented in Equation (9).

Complementary to water permeation, after measuring water vapor sorption kinetics of the multilayer film samples, the corresponding water vapor sorption isotherms were plotted and are presented in Figure 11. For comparison, water sorption isotherm of PLA and PBSA were also plotted in Figure 11. First, it can be seen that PLA and PBSA water sorption behaviors are similar and characteristic of Henry mode sorption (linear curve) with a low water sorption capacity not exceeding 1% and slightly higher for PBSA. In general, the PLA/PBSA multilayer have a linear increase of mass gain with the water activity comparable to PLA, except for PLA/PBSA film from *a_w_* > 0.5, showing a minor water clustering phenomenon. If we can observe that the water sorption capacity of multilayer films is governed by the major phase of PLA, it is however surprising to see that higher the presence of clays, lower the mass gain (in opposite to PBSA composites, Figure 5) and the presence of water aggregation in PLA/PBSA0 multilayer which is not so pronounced for PLA and PBSA. This result is clearly opposite to the sorption behavior of the filled PBSA films where the hydrophilic contribution of clays in PBSA matrix has induced a water uptake. In the case of multilayer films, the sorption curve profile indicates a reduction in water clustering. We can infer that the C30B acts as obstacles to water diffusion by tortuosity effects. The contribution of C30B to re-up the water mass gain at higher water activities due to water-sensitive groups is stopped by the layered microstructure. This effect is also a function of filler content. The confinement to filled PBSA layers by amorphous PLA layers prevents an increase in water solubility. The layer breaks in multilayer films as observed in TEM micrographs have not altered the barrier contribution of clays for water vapor diffusing molecules. 

The combination of two sorption modes—Henry-law and aggregation modes—was applied to simulate the isotherm curves and the modelling parameters are summarized in Table 8. The MDM values lower than 10% testifies to the appropriate fitting of sorption data. The *k_D_* parameter can be indicated as a constant value for the three multilayer films. So, the water solubility is not impacted by the C30B incorporation within the layered structure. The reduction in water clustering formation is clearly evidenced from the variation of *K_a_* and *n* values. With the C30B content increase, the aggregation reaction is reduced and the water cluster size is largely impacted. In the case of vapor sorption, the preferential diffusion pathways occurring at the clay-polymer interfacial areas, as discussed from permeation data, seem to be blocked by the polymer alternating layers structure of films. The modelling approach is correlated with the sorption isotherms. The water plasticization effect is, as a consequence, limited due to the reduced water concentration. As the degree of crystallinity is not really changed, one can report this result to increase stiffness and to the RAF increase, which has impacted the local macromolecular chains rigidity at the clay-polymer interfacial areas. Also, we can suppose that a specific orientation of PBSA crystals induced by the confinement effect in multilayer could hinder the passage of water molecules. From a previous study on PLA/PBSA multilayer films without fillers [18], it was shown from WAXS patterns recorded on the face and on the cross section a slight crystal orientation on both transverse and extrusion view patterns. Thus, it is possible that in filled PLA/PBSA multilayers, this slight orientation is still present, as the filler content is relatively low. 

**Figure 12:**
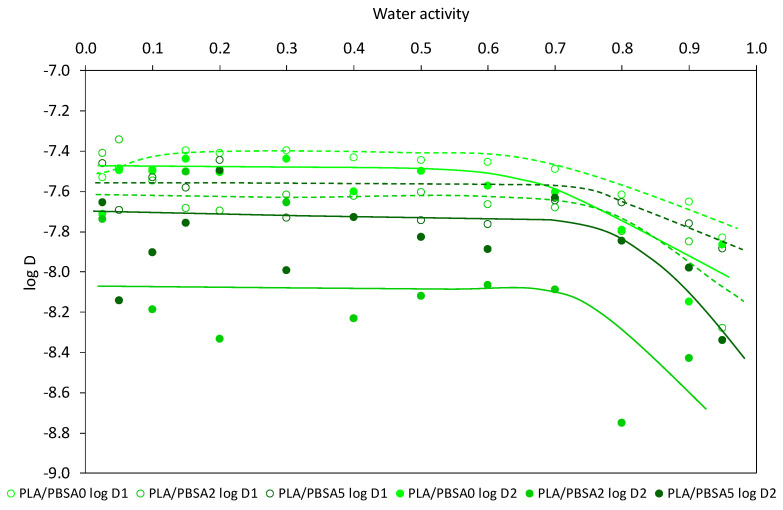
Water vapor diffusion coefficients *D*_1_ and *D*_2_ for the PLA/PBSA, PLA/PBSA2 and PLA/PBSA5 multilayer films.

The diffusivity behavior in sorption measurement for the PLA/PBSA multilayer films was also evaluated in a semi-logarithmic scale (log *D*) as a function of water activity, as presented in Figure 12. The diffusion curve profiles are rather similar for all the films, irrespective of the *D* coefficient. As for PBSA nanocomposites, in unfilled and filled PLA/PBSA multilayers, a decrease in diffusion is observed at high water activities due to the water clustering phenomenon, even though this phenomenon is not so significant. Again, like for the PBSA-based films, the *D*_2_ coefficients are found to be lower than the *D*_1_ coefficients in the whole range of water activity. The balance between increase and reduction of water diffusion is again considered to explain this curve profile. Regarding the variation of *D*_1_ and *D*_2_ coefficients as C30B content increases, one can note that both *D*_1_ and *D*_2_ coefficients are reduced, highlighting tortuosity effects induced by C30B on water diffusion at the film surface and in the core of the film, respectively. In addition to the tortuosity effect, geometrical constraints and reduced chain segment mobility contribute to water mobility restriction. 

For this second part of the work, one can infer that the incorporation of C30B fillers within PBSA layers of PLA/PBSA multilayer film has induced an increase of the glass transition temperature, while the degree of crystallinity and RAF is unchanged or slightly for PLA/PBSA2, showing less chain mobility by confinement effect and a local rigidification of chains by close clay contact in a thin layer thickness (150 nm on average). However, wider thicknesses for the filled PBSA layers are obtained compared to the neat PBSA layers in the layered structure due to the clay presence. In addition, some defects as layer breaks are observed without polymer interdiffusion at the polymer-polymer interfaces. From TEM micrographs; clays are located in the PBSA layers or at the polymer-polymer interfaces without an effect on PLA mobility since glass transition is unchanged. 

One can point out an additional improvement of water barrier performances by tortuosity effects compared to the behaviour of the neat PLA/PBSA multilayer film. By permeation, a shift of the water flux curves to lower time is measured indicating a faster water diffusion through preferential diffusing pathways generated at the polymer-C30B interfacial areas while by sorption a decrease in *D* coefficients is measured evidencing greater difficulty in forming water clusters and their diffusion in the multilayer films due to geometrical constraints and chain rigidification. 

It should be remembered that the boundary conditions are different between water permeation and water vapor sorption measurements. For water permeation, only one side of the tested film is in contact with liquid water and the water diffusion is deduced from the quantity of water molecules passing through the film thickness, while for water vapor sorption both sides of the film are in contact with water vapor and the water diffusion results from water molecules penetrating the film in opposite directions inside the tested film. Therefore, the duration of the measurement is longer for sorption process carried out at various water activities compared with the permeation process. It seems that the formation of water clusters is considered a slow phenomenon and that leads to reduce water mobility (decrease of *D*) has not sufficient time to occur in permeation as it can be clearly observed with water sorption. 

## 4. Conclusions

In this work, we successfully prepared biodegradable PLA/PBSA multinanolayer films loaded with lamellar nanoclays in confined PBSA layers. It was observed that the incorporation of the organo-modified montmorillonite led to some layer breakups, especially in the presence of aggregates. As a consequence, irregularities in the thickness of layers (which doubled or tripled compared to the unfilled PLA/PBSA multilayer film) may appear. Despite this, the integrity and cohesion of the loaded multilayered structure was overall maintained, leading to the same mechanical and water barrier properties as the unfilled multilayer. Besides, we have shown that inclusion of clays in PBSA (monolayer comparing or nanolayer confined by PLA layers in a multilayer structure) leads to an improvement of the water barrier properties. The highest improvement factor (IF ~ 58%) was obtained when fillers are oriented and confined in very thin layers and for the highest filler content. However, this improvement is not as high as expected, probably because of the presence of some defects and aggregates and the absence of impact on the crystallinity. Consequently, we consider that these biodegradable PLA/PBSA multilayer films loaded with lamellar clays are promising materials. Our next goal will be to investigate the impact on barrier properties of a subsequent stretching step, and of a higher filler content. 

## Figures and Tables

**Table 1:** Thermal properties of the neat filled PBSA films.

	*T_g_* PBSA (°C)	*T_c_* PBSA (°C)	*T_m_* PBSA (°C)	Δ*C_p_* PBSA (J g^−1^ °C^−1^)	*X_c_* PBSA (%)	RAF (%)
**PBSA**	−46	67	90	0.330	38 ± 2	9 ± 3
**PBSA2**	−43	66	91	0.275 *	38 ± 2	17 ± 3
**PBSA5**	−42	66	91	0.274 *	37 ± 2	18 ± 3

* The value of Δ*C_p_* is calculated taking into account the proportion of PBSA in the composite.

**Table 2:** Influence of the filler content on the mechanical properties of PBSA film.

	Young’s Modulus (MPa)	Strength at Break (MPa)	Elongation at Break (%)
**PBSA**	241 ± 19	35 ± 3	1360 ± 148
**PBSA2**	276 ± 33	31 ± 2	1405 ± 67
**PBSA5**	362 ± 19	27 ± 2	1242 ± 97

**Table 3:** Water permeation parameters for the PBSA, PBSA2 and PBSA5 films.

	P (Barrer *)	*D*_0_ (10^−8^ cm^2^·s^−1^)	*D_M_* (10^−8^ cm^2^·s^−1^)	*γC_eq_*	*γ* (cm^3^·mmol^−1^)	*C_éq_* (mmol·cm^−3^)
**PBSA**	8312 ± 177	1.8 ± 0.1	36 ± 3	3.0 ± 0.1	3.9 ± 0.4	0.78 ± 0.05
**PBSA2**	5628 ± 127	1.7 ± 0.1	25 ± 6	2.7 ± 0.3	3.9 ± 0.9	0.69 ± 0.09
**PBSA5**	4821 ± 189	1.5 ± 0.1	15 ± 1	2.3 ± 0.1	2.7 ± 0.3	0.85 ± 0.03

* 1 Barrer = 10^−10^ cm^3^ (STP) cm·cm^−2^·s^−1^·cmHg^−1^.

**Table 4:** Modelling parameters for the neat PBSA and the filled PBSA films.

	*k_D_g*/*g*	*n*	*K_a_* (*g*/*g*)^(1−*n*)^	MDM (%)
**PBSA**	0.583	1.4	0.407	5.10
**PBSA2**	0.582	2.6	0.803	5.54
**PBSA5**	0.702	3.1	0.811	5.56

**Table 5:** Thermal properties of the neat and filled PLA/PBSA multilayer films.

	*T_g_* PBSA (°C)	*T_g_* PLA (°C)	*T_c_* PBSA (°C)	*T_m_* PBSA (°C)	Δ*C_p_* PBSA (J g^−1^ °C^−1^)	*X_c_* PBSA (%)	RAF (%)
**PLA/PBSA (PBSA thickness ~60 nm)**	−46	53	71	91	0.250 *	40	21
**PLA/PBSA2 (PBSA thickness ~100 nm)**	−42	54	71	90	0.204 *	34	33
**PLA/PBSA5 (PBSA thickness ~200 nm)**	−36	54	71	91	0.237 *	38	23

* The value of Δ*C_p_* is calculated given the proportion of PBSA in the multilayer film.

**Table 6:** Mechanical parameters for the multilayer film PLA/PBSA films.

	Young’s Modulus (MPa)	Strength at Break (MPa)	Elongation at Break (%)
**PLA/PBSA**	1723 ± 79	38 ± 3	37 ± 10
**PLA/PBSA2**	1541 ± 61	34 ± 2	55 ± 12
**PLA/PBSA5**	1686 ± 66	38 ± 3	39 ± 7

**Table 7:** Water permeation parameters for the PLA/PBSA, PLA/PBSA2 and PLA/PBSA5 multilayer films. Experimental and predicted values.

	Water Permeability (Barrer)	Calculated Permeability (Series Model) (Barrer)	Calculated P of PBSA Layers (Barrer)	*D*_0_ (10^−8^ cm^2^·s^−1^)	*D_M_* (10^−8^ cm^2^·s^−1^)	*γC_eq_*	*γ* (cm^3^·mmol^−1^)	*C_eq_* (mmol·cm^−3^)
**PLA/PBSA**	2765 ± 123	2917	4658	1.48 ± 0.04	12.7 ± 0.4	2.1 ± 0.1	3.8 ± 0.1	0.56 ± 0.01
**PLA/PBSA2**	2717 ± 136	2917/2823 *	4055	1.61 ± 0.06	16.0 ± 1.0	2.3 ± 0.1	5.1 ± 0.6	0.47 ± 0.05
**PLA/PBSA5**	2659 ± 81	2917/2776 *	3487	1.71 ± 0.1	13.0 ± 1.1	2.0 ± 0.2	4.1 ± 0.6	0.50 ± 0.05
**PLA monolayer**	2510 ± 177			0.87 ± 0.07	11.6 ± 0.7	2.6 ± 0.2	4.0 ± 0.1	0.67 ± 0.03
**PBSA monolayer**	8312 ± 177			1.8 ± 0.1	36 ± 3	3.0 ± 0.1	3.9 ± 0.4	0.78 ± 0.05

* Calculated from loaded PBSA monolayer (Table 3).

**Table 8:** Modelling parameters for the PLA/PBSA, PLA/PBSA2 and PLA/PBSA5 multilayer films.

	*k_D_g*/*g*	*n*	*K_a_* (*g*/*g*)^(1−*n*)^	MDM (%)
**PLA/PBSA**	0.677	3.83	0.209	3.25
**PLA/PBSA2**	0.614	0.64	0.109	3.65
**PLA/PBSA5**	0.621	0.06	0.012	4.87
